# Videodensitometric analysis of advanced carotid plaque: correlation with MMP-9 and TIMP-1 expression

**DOI:** 10.1186/1476-7120-9-24

**Published:** 2011-09-18

**Authors:** Liz Andréa V Baroncini, Antonio Filho Pazin, Luiz Otávio Murta Junior, Lia S Nakao, Simone G Ramos, Dalton B Précoma

**Affiliations:** 1 Department of Health and Scienses - Pontificia Universidade Católica do Paraná, Rua Imaculada Conceição 1155, Curitiba, Paraná, CEP: 80215901, Brazil; 2 Division of Emergency Medicine, Faculdade de Medicina de Ribeirão Preto, USP, Rua Bernardino de Campos 1000, Ribeirão Preto, São Paulo, CEP: 14015130, Brazil; 3 Department of Computer Scienses and Mathematics, FFCRP, USP, Avenida Bandeirantes 3900, Ribeirão Preto, São Paulo, CEP: 14040901, Brazil; 4 Department of Basic Pathology, Universidade Federal do Paraná, Centro Politécnico, Curitiba, Paraná, CEP: 80531980, Brazil; 5 Department of Pathology, Faculdade de Medicina de Ribeirão Preto, USP, Avenida Bandeirantes 3900, Ribeirão Preto, São Paulo, CEP: 14049900, Brazil

**Keywords:** carotid arteries, atherosclerosis, ultrasound tissue characterization

## Abstract

**Background:**

Matrix metalloproteinase-9 (MMP-9) and tissue inhibitor of MMP (TIMP) promote derangement of the extracellular matrix, which is ultimately reflected in plaque images seen on ultrasound. Videodensitometry can identify structural disturbances in plaques.

**Objectives:**

To establish the correlations between values determined using videodensitometry in B-mode ultrasound images of advanced carotid plaques and the total expression of MMP-9 and TIMP-1 in these removed plaques.

**Methods:**

Thirty patients underwent ultrasonic tissue characterization of carotid plaques before surgery, using mean gray level (MGL), energy, entropy and homogeneity. Each patient was assigned preoperatively to one of 2 groups: group I, symptomatic patients (n = 16; 12 males; mean age 66.7 ± 6.8 years), and group II, asymptomatic patients (n = 14; 8 males; mean age 67.6 ± 6.81 years). Tissue specimens were analyzed for MMP-9 and TIMP-1 expression. Nine carotid arteries were used as normal tissue controls.

**Results:**

MMP-9 expression levels were elevated in group II and in normal tissues compared to group I (p < 0.001). TIMP-1 levels were higher in group II than in group I, and significantly higher in normal tissues than in group I (p = 0.039). The MGL was higher in group II compared to group I (p = 0.038). Energy had greater values in group II compared to group I (*p *= 0.02). There were no differences between patient groups in homogeneity and entropy. Energy positively correlated with MMP-9 and TIMP-1 expression (p = 0.012 and p = 0.031 respectively). Homogeneity positively correlated with MMP-9 and TIMP-1 expression (p = 0.034 and p = 0.047 respectively). There were no correlations between protein expression and MGL or entropy.

**Conclusions:**

Videodensitometric computer analysis of ultrasound scanning images can be used to identify stable carotid plaques, which have higher total expression levels of MMP-9 and TIMP-1 than unstable plaques.

## Background

Advanced carotid plaques are complex structures, and their classification remains a challenging problem. It is not currently possible to predict whether a carotid plaque will produce symptoms or when symptoms will occur [1]. The American Heart Association classification of human atherosclerotic lesions considers the presence or absence of different tissue components in plaques, but does not account for the proportion of each component or its location in the plaques [2]. Because plaques are dynamic structures, changing through processes of stabilization and destabilization, the locations and the proportions of different plaque components are constantly being modified. Matrix metalloproteinase 9 (MMP-9) and tissue inhibitor of MMP (TIMP-1), which are secreted by infiltrating macrophages and smooth muscle cells, are present in all stages of atherosclerotic plaque progression, from normal tissue to advanced lesions, and are factors involved in plaque remodeling. These proteins promote the derangement of extracellular matrix, which is ultimately reflected in plaque images seen on Doppler ultrasound [3,4]. Carotid plaques evaluated by ultrasound are usually described in terms of echogenicity, homogeneity, board regularity, ulceration, localization, extension, and the degree of stenosis. The final decision for surgical intervention is primarily based on the degree of plaque stenosis and on the patient symptoms. Previous studies have attempted to correlate the histological composition of surgically removed carotid plaques and their ultrasonic characteristics as determined by presurgical bidimensional imaging [5-8]. In a previous study from our laboratory, we were able to use videodensitometry to correlate the echogenicity of carotid images with the fibrous tissue and lipid components of removed specimens [9]. We established that the first order parameter mean gray level determined from videodensitometric computer analysis of scan images may be used to identify vulnerable and potentially unstable lipid-rich carotid plaques, which are less echogenic in density than stable or asymptomatic, more densely fibrotic plaques. In that same study, we were able to differentiate stable from vulnerable plaques based on second-order parameter energy, although there were no correlation with the amounts of fibrous tissue or lipid components. We believe that videodensitometric second order parameters may be useful in identifying structural derangements in plaques, including inflammatory processes and disrupted extracellular matrix, that are modulated by matrix metalloproteinases and their inhibitors. We choose MMP-9 and TIMP-1 as markers of structural derangement in order to evaluate matrix remodeling in surgically removed carotid plaques. These proteins have been well studied and their role in plaque destabilization has been established. The objectives of the present study were to correlate the results of statistical analysis of videodensitometric parameters with MMP-9 and TIMP-1 expression levels in advanced carotid plaques.

## Methods

### A. Patients

Thirty nonconsecutive surgical inpatients (20 males, mean age 68.03 ± 7.3 years) admitted for carotid endarterectomy for extracranial high-grade (^3 ^70%) internal carotid artery stenosis were selected for this study. The only inclusion criterion was that the patient should be already scheduled for surgery based on the degree of carotid stenosis and the presence of symptoms. If the patient was asymptomatic with stenosis > 70%, the indication for surgical therapy depended on comorbidities and vertebro-basilar (in)sufficiency. Exclusion criteria were the presence of suboptimal ultrasonographic visualization of the atherosclerotic plaque contour/border, or surgical specimen inadequate for immunocytochemical analysis. Institutional ethical committee approval was obtained for the study and for procurement of specimens. Written informed consents were obtained from all patients. A clinical history and examination were performed which included a neurological exam to establish the number and duration of ischemic events. Before surgery, all patients underwent the following: (1) either cerebral angiography or magnetic resonance angiography plus Duplex ultrasound to grade carotid artery stenosis and assess the intracranial arterial system; and (2) either computed tomography (CT) or magnetic resonance imaging (MRI) to examine the brain. The presence or absence of infarction in the corresponding middle cerebral artery territory was noted. Focal cerebral ischemic events were identified as transient ischemic attack (TIA), amaurosis fugax (AF), central retinal artery occlusion, or cerebrovascular accident. Patients were considered to be symptomatic if they had experienced AF, TIA, or stroke ipsilateral to the carotid lesion being studied. Patients without any history of recent neurologic symptoms or with nonspecific, nonhemispheric symptoms such as dizziness and vertigo were considered asymptomatic. Each patient was assigned preoperatively to one of 2 groups based on symptoms: group I consisted of symptomatic patients (n = 16; 12 males; mean age 66.7 ± 6.8 years), and group II consisted of asymptomatic patients (n = 14; 8 males; mean age 67.6 ± 6.81 years). Baseline determination of each patient consisted of the following: height, weight, body mass index, blood pressure, fasting serum total cholesterol (TC), high density lipoprotein cholesterol (HDL-C), low density lipoprotein cholesterol (LDL-C), triglycerides (TGC), fasting plasma glucose, electrocardiogram, and information regarding history of coronary artery disease, diabetes mellitus, and smoking habits. There were no substantial differences between the 2 patient study groups in percent reduction of carotid diameter, procedural surgical methods, concomitant therapy, age, sex, and risk factors (Table 1).

**Table 1:** Baseline patient characteristics

	Group I (n = 16)	Group II (n = 14)
Age, years	66.7 ± 6.8	67.6 ± 6.8
Sex, M/F	12/4	8/6
Hypertension	10	1
Diabetes Mellitus	2	3
Active smoking	3	3
Hypercholesterolemia	2	1
CAD	4	0
Aspirin	15	10
Statin	5	4
ACE inhibitors	9	8
Ticlopidine	4	1

### B. Tissue specimens and immunoblotting

Carotid plaques were obtained immediately after endarterectomy. All surgeries were performed using standard surgical techniques and with minimal manipulation of the specimen. All plaques were removed en bloc, without fragmentation or significant distortion. Sections used for MMP-9 and TIMP-1 quantification were snap-frozen in liquid nitrogen and stored at -70°C until processing. The protein expression levels of MMP-9 and TIMP-1 in tissue extracts were analyzed by immunoblotting following sodium dodecyl sulfate polyacrylamide gel electrophoresis (SDS-PAGE). Specific regions of each plaque were not preselected for this analysis. The section was analyzed entirely, as described previously [2]. Briefly, carotid samples (n = 30) were minced and homogenized in lysis buffer containing PBS (pH 7.2), 0.5% Triton X-100, 0.1% SDS, 0.5% sodium deoxycholate, and a protease inhibitor cocktail (Roche) at 4°C. The extracts were centrifuged (5000 rpm, 15 min, 4°C), and supernatants were collected and stored at -20°C until analysis. Total protein concentrations in the lysates were determined using the Bradford protein assay. Proteins (10 μg) were separated through a 10% acrylamide/bisacrylamide SDS gel and transferred to a nitrocellulose membrane. After incubation in nonfat milk, membranes were probed with anti-MMP (1:1000), or anti-TIMP-1 (1: 50) antibodies (Santa Cruz Biotechnology), followed by incubation in secondary antibodies conjugated to horseradish peroxidase (HRP). Reactions were developed using SuperSignal WestPico Chemiluminescent Substrate (Thermo Fisher Scientific Inc., Rockford, IL, USA). For endogenous controls, membranes were also probed with anti-β actin (1:5000; Sigma-Aldrich, St. Louis, MO, USA) and anti-mouse IgG conjugated to HRP (KLP 1:2000; Kirkegaard and Perry Laboratories, Gaithersburg, MD, USA). Densitometry analysis was performed using ImageJ software. Nine carotid arteries were removed from human adult cadavers that had not been preselected for carotid artery disease. These specimens did not have any macroscopic signs of atherosclerotic plaques and were used as normal tissue controls.

### D. Ultrasonographic Image Acquisition and Preprocessing

Ultrasound assessment was performed 1 to 2 days before carotid endarterectomy. Conventional echo images were acquired with a commercially available 2D ultrasonic imaging system (Hewlett-Packard Sonos 5500; Andover, MA, USA). The system characterized arterial tissue at the bedside using a 5- to 12- MHz multifrequency linear transducer for all studies. This software enables the acquisition, storage, and retrieval of a sequence of continuous 2D conventional images, forming a continuous-loop digital recording of 2 s (60 frames per 2 s). Anterior, lateral, and posterior projections were used to image the plaque longitudinally. The position of the probe was adjusted so that the ultrasonic beam was vertical to the artery wall. Offline analysis of the 2D images was performed after retrieving the stored data from the built-in optical disc drive in the system. For videodensitometric analysis, the images from the magnetic optical disk were loaded into a computer installed with a specific software program (CaPAS - Carotid Plaque Analysis Software; University of Sao Paulo, Sao Paulo, Brazil). Subjective selection was performed to optimize the contours/borders, area, and contrast of each plaque. Selected static frames considered appropriate for analysis fulfilled the following criteria: 1) blood in the vicinity of the plaques was dark and echoically uniform, and 2) the atherosclerotic plaque was well delineated, horizontal, and of maximum thickness.

### E. Quantitative texture analysis

**Figure 1:**
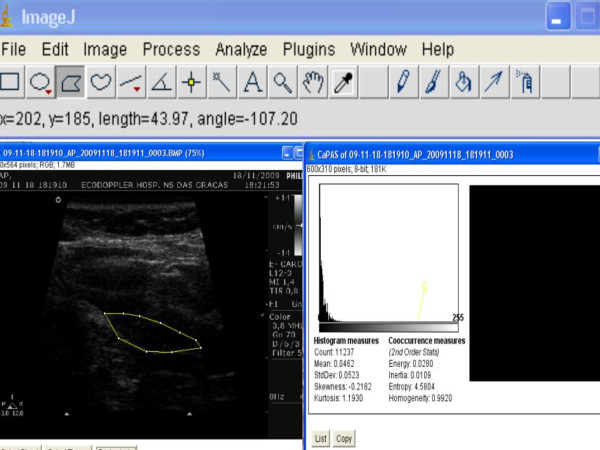
**Hypoechoic (lipid) carotid plaque and CaPAS analyzes**. Left panel: bidimensional image of carotid plaque. Right panel: CaPAS parameters

**Figure 2:**
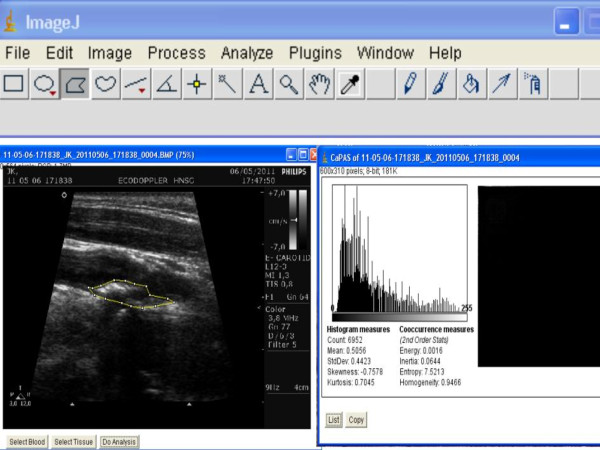
**Hyperechoic (fibrotic) carotid plaque and CaPAS analyzes**. Left panel: bidimensional image of carotid plaque. Right panel: CaPAS parameters

All plaque images were evaluated by CaPAS software for texture parameters, which include a set of first-order (mean gray level) and of second-order (entropy, energy, and homogeneity) parameters. These parameters were described in a previous study [9]. Briefly, the mean gray level (MGL) represented the median of the frequency distribution of gray tones of the pixels included in the region of interest (ROI) (gray-scale median of the region) for a scale of 256 gray tones (0 = darkest tone, 255 = brightest tone) [10]. Dark (hypoechoic) regions were associated with a gray-scale median (GSM) that tended to approach 0, whereas bright (hyperechoic) regions were associated with a GSM that tended to approach 255. The energy or angular second-moment value increases when elements in the co-occurrence matrix are very unequal. Entropy and homogeneity reflect the degree of coarseness of the image, as its value increases when homogeneity is reduced; i.e., when elements in the co-occurrence matrix tend to be equal and the diagonal concentration lowers. The mathematical definitions of these texture parameters have been described in a previous article [8]. Plaque images were normalized using 2 echo-anatomic reference points: the GSM of the blood and the GSM of the periadventitial region. After normalization, each image was manually outlined in its longitudinal section 3 times by the same examiner. The mean score of these 3 sequential measurements was used for analysis (Figures 1 and 2). Examiner performing the ultrasonography and outlining the images was blinded to the results of immunohistochemical analysis.

#### Statistical analysis

Categorical variables were expressed as percentages, and continuous variables were expressed as means ± SD and medians. The Shapiro-Wilk test was used to evaluate sample normality. For quantitative parameters, the Mann-Whitney nonparametric test was used to compare groups. The Spearman coefficient was used to identify correlations between quantitative variables. Statistical significance was indicated by a value of *p *< 0.05. Analyses were performed using Statistica v. 8.0. Intra- and inter-examiner variabilities in ultrasonographic measurements were tested for all carotid images as proposed by Lin [10].

## Results

### A. Expression of MMP-9 and TIMP-1

MMP-9 expression was significantly elevated in group II and in normal tissues compared to group I (p < 0.001). TIMP-1 levels were higher in group II and in normal tissues than in group I, with statistical difference between normal tissue and GI (p = 0.039; Table 2).

**Table 2:** MMP-9 and TIMP-1 expression (in arbitrary units of band densitometry, normalized by β-actin expression), in the study groups

Variable	Group	Mean	Median	SD	p value
MMP-9	GI	127	147	44.3	
	GII	198.9	201.3	11.5	< 0.001
	Control	182.1	181	25.2	
TIMP-1	GI	1.2	0	1.8	
	GII	2.1	2.1	1.8	0.039
	Control	3.6	2.4	2.7	

MMP-9 means metalloproteinase-9

TIMP-1 means tissue inhibitor of metalloproteinase-1

### B. Videodensitometric Analysis

Among first order-parameters, MGL had higher values in group II (p = 0.038). Among second order-parameters, energy distinguished group I from group II with higher values in group II (*p *= 0.012). There were no significant differences for homogeneity and entropy between groups (Table 3).

**Table 3:** Comparisons of videodensitometric parameters between groups I and II

Variables		N	Mean	Median	SD	p-value*
MGL	GI	16	0.390	0.400	0.126	
	GII	14	0.505	0.556	0.159	**0.038**
Entropy	GI	16	5.676	5.706	0.390	
	GII	14	5.531	5.636	0.496	0.423
Energy	GI	16	0.006	0.005	0.004	
	GII	14	0.024	0.010	0.028	**0.012**
Homogeneity	GI	16	0.201	0.195	0.043	
	GII	14	0.236	0.206	0.072	0.166

* Mann-Whitney non-parametric test, p < 0,05 considered significant

### C. Correlation Between Immunoblotting and Videodensitometric Analysis

**Figure 3:**
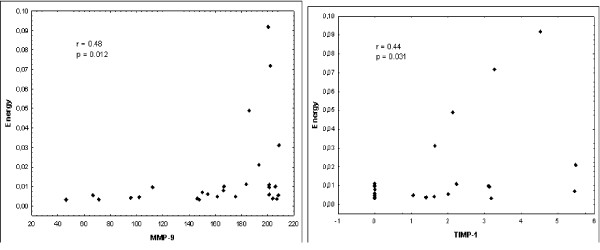
**Correlations between protein expressions levels and energy parameters**.

**Figure 4:**
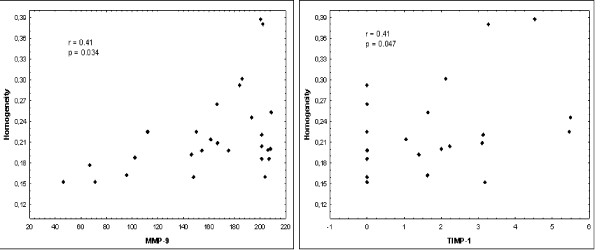
**Correlations between protein expressions levels and homogeneity parameters**.

Energy, a second-order parameter, positively correlated with MMP-9 and TIMP-1 expression (p = 0.012 and p = 0.031 respectively). Homogeneity, a second-order parameter, also positively correlated with MMP-9 and TIMP-1 expression (p = 0.034 and p = 0.047 respectively). There was no statistically significant correlation between MGL or entropy and protein expression levels (Table 4; Figures 3 and 4).

**Table 4:** Correlations between protein expressions levels and videodensitometric parameters

Variable		n	Correlation coefficient	p value
MMP-9 versus	MGL	30	0.18	0.357
	Entropy	30	-0.30	0.133
	Energy	30	0.48	**0.012**
	Homogeneity	30	0.41	**0.034**
TIMP-1 versus	MGL	30	0.10	0.638
	Entropy	30	-0.34	0.102
	Energy	30	0.44	**0.031**
	Homogeneity	30	0.41	**0.047**

## Discussion

The ultrasound imaging description of a plaque requires more than reporting the degree of stenosis. In evaluating the echogenicity of a carotid plaque, the ultrasonographer visualizes the plaque as a unique structure, and s/he can only subjectively determine its characteristics in general terms (i.e., hypoechoic or hyperechoic, heterogeneous or homogeneous). These characteristics are too subjective for daily clinical practice and are affected by the training and experience of the ultrasonographer. Despite advances in duplex ultrasound scanning technology, the process of acquiring, analyzing, and interpreting B-mode images has remained observer-dependent. Because various classifications have relied on the human observer rather than objective measurements, there has been great variability in the incidence of cerebrovascular events in relation to plaque morphology. A previous investigation [11] based on the appearance of radiofrequency signal determined that the echo coming from the transition between the intima-media arterial wall was of relatively low amplitude in normal and fatty samples compared to others pathologic subsets. This pioneering study from Picano et al. has been a great contribution to the field of ultrasound image analysis and image correlation with different tissues. Therefore, correlating plaque proteins expression with ultrasonic textural characteristics of carotid plaques based on videodensitometry, may be a noninvasive quantitative method for the identification of vulnerable carotid plaques. The videodensitometric method analyzes the echogenic and ultrasonic characteristics of the entire image selected. It reports the parameters obtained from the analyses of the entire selected image and can assess the extent of structural disorder represented by the image. In the present study, we correlated parameters from videodensitometric analyses with protein expression in plaques. In our previous study [2], we determined that expression levels of MMP-9 and TIMP-1 were higher in surgical specimens from asymptomatic patients, which corresponded to more stable chronic plaques. In that study, the entire section of plaque was analyzed and the total expression of each protein was determined. Another study [12] has corroborated these findings, and the authors speculated that a certain baseline level of metalloproteinase activity is necessary for the maintenance of a stable carotid plaque. Therefore, in the present study, we assessed the videodensitometric parameters acquired from the total imaging depicted and correlated them with the results from the entire section of plaque that was analyzed. We found positive correlations for MGL, homogeneity, and energy with expressions levels of MMP-9 and TIMP-1. Among first-order parameters, MGL assess overall contrast and the echodensity of an image and correlate well with stable or fibrotic and calcified carotid plaques [8,13-24]. First-order statistical parameters are mathematical descriptors of the shape of the frequency distribution of a gray-level histogram, and are used to describe the overall brightness of the ROI. Classically, they correlate with structural tissue components based on attenuation of the ultrasound beam, and represent the intensity of image brightness without determining regional variations in the plaque. The second-order parameters are associated with patterns of texture, and depend less on the intensity of brightness and more on heterogeneity of the image. They are mathematical descriptors of the spatial distribution and dependence of gray levels within the ROI. They are based on a co-occurrence matrix that estimates the probability that a pair of pixels, each with its own gray level and separated by a displacement vector, occurs in the considered ROI. The second-order parameters do not indicate the amount of a specific tissue component, but how it is arranged and organised spatially. Therefore, in the present study, we attempted to correlate images derangements to protein expression levels. In that sense, the metalloproteinases are involved in the remodelling processes of the plaque [25-31]. According to our findings, MMP-9 and TIMP-1 were expressed at higher levels in normal tissues and in surgical specimens from asymptomatic patients than in specimens from symptomatic patients. Secreted by macrophages and smooth muscle cells in attempts to stabilize the plaque, MMP-9 and TIMP-1 derange the extracellular matrix and cause imbalances. Finally, attempting to correlate videodensitometric parameters with specific regions inside the plaques, such as regions with increased concentrations of inflammatory cells or highly vulnerable regions like the shoulder of a plaque, does not seem to be feasible. At the present time, it is not possible to use bidimensional imaging to select a specific region in the image and correlate that with immunohistochemical parameters. Bidimensional ultrasound produces an image of gray tone variations and it is impossible for the human eye to distinguish specific or small regions inside the plaque. While magnetic resonance (MRI) can be used to discriminate between the different stages of thrombus and hemorrhage formation, the videodensitometric approach with bidimensional images can not distinguish intra-plaque hemorrhage or classify plaque subtypes [32,33]. Therefore, we assessed the image of an entire plaque and attempted to correlate with the entire section of the surgically removed plaque. Based on our results, plaques identified in bidimensional images as having higher energy values and greater homogeneity should be more stable and less prone to rupture. Such knowledge would change the clinical approach taken to treat to the patient. Using an inexpensive method, a physician could determine whether an asymptomatic patient with a high-degree of carotid stenosis needed immediate surgical treatment. In addition, since plaques can be easily assessed using this method, they can be frequently evaluated for changes in their structure. However, the videodensitometric approach, like radiofrequency analysis, is still limited to research applications and requires time and analysis away from the echo machine. We believe that this is the first study to use this approach and that future studies are needed with more patients to corroborate our findings.

## Conclusions

Videodensitometric computer analysis of ultrasound scanning images may be used to identify more stable carotid plaques, which have higher expression levels of MMP-9 and TIMP-1 than unstable plaques.

## Competing interests

The authors declare that they have no competing interests.

## Authors' contributions

LAVB, APF and DBP design the study and wrote the manuscript. LOMJ developed the CaPAS software. LSN and SGR analyzed the surgical specimens. All authors read and approved the final manuscript.
